# Single Nucleotide Polymorphism Detection for Peach Gummosis Disease Resistance by Genome-Wide Association Study

**DOI:** 10.3389/fpls.2021.763618

**Published:** 2022-02-07

**Authors:** Xiongwei Li, Jiabo Wang, Mingshen Su, Jingyi Zhou, Minghao Zhang, Jihong Du, Huijuan Zhou, Kexin Gan, Jing Jin, Xianan Zhang, Ke Cao, Weichao Fang, Lirong Wang, Huijuan Jia, Zhongshan Gao, Zhengwen Ye

**Affiliations:** ^1^Forest and Fruit Tree Institute, Shanghai Academy of Agricultural Sciences, Shanghai, China; ^2^Key Laboratory of Qinghai-Tibetan Plateau Animal Genetic Resource Reservation and Utilization (Southwest Minzu University), Ministry of Education, Chengdu, China; ^3^Horticultural Department, Shanghai Municipal Agricultural Technology Extension and Service Center, Shanghai, China; ^4^Key Laboratory for Horticultural Plant Growth, Department of Horticulture, Development and Quality Improvement of State Agriculture Ministry, Zhejiang University, Hangzhou, China; ^5^Zhengzhou Fruit Research Institute, Chinese Academy of Agriculture Sciences, Zhengzhou, China

**Keywords:** peach, gummosis disease, QTLs, genome-wide association study, candidate genes

## Abstract

Peach gummosis is one of the most widespread and destructive diseases. It causes growth stunting, yield loss, branch, trunk, and tree death, and is becoming a restrictive factor in healthy and sustainable development of peach production. Although a locus has been identified based on bi-parental quantitative trait locus (QTL) mapping, selection of gummosis-resistant cultivars remains challenging due to the lack of resistant parents and of the complexity of an inducing factor. In this study, an integrated approach of genome-wide association study (GWAS) and comparative transcriptome was used to elucidate the genetic architecture associated with the disease using 195 accessions and 145,456 genome-wide single nucleotide polymorphisms (SNPs). The broad-sense and narrow-sense heritabilities were estimated using 2-year phenotypic data and genotypic data, which gave high values of 70 and 73%, respectively. Evaluation of population structure by neighbor-joining and principal components analysis (PCA) clustered all accessions into three major groups and six subgroups, mainly according to fruit shape, hairy vs. glabrous fruit skin, pedigree, geographic origin, and domestication history. Five SNPs were found to be significantly associated with gummosis disease resistance, of which SNPrs285957, located on chromosome6 across 28 Mb, was detected by both the BLINK and the FarmCPU model. Six candidate genes flanked by or harboring the significant SNPs, previously implicated in biotic stress tolerance, were significantly associated with this resistance. Two highly resistant accessions were identified with low disease severity, which could be potential sources of resistance genes for breeding. Our results provide a fresh insight into the genetic control of peach gummosis disease.

## Introduction

Peach [*Prunus persica* (L.) Batsch] is one of the most economically important deciduous fruit from the Rosaceae family (Li et al., 2013). It originated in northwest China, and has spread throughout China and the rest of the world because of its greater adaptability (Faust and Timon, 1995; Yu et al., 2018). But, the short-life syndrome due to gummosis is a long-lasting problem in the warm and moist climate regions.

Gummosis is a nonspecific disease response to pathogen infection, mechanical injury, drought and cold stress, or insect attack. It is characterized by a gum exudation from tree trunks, branches, and fruits in several fruit species, such as peach (Britton and Hendrix, 1982), almond (Popović et al., 2021), apricot (Liu et al., 2015), sweet cherry (Zhang L. et al., 2019), and in citrus (Fan et al., 2011). Gummosis in peach was first reported in central Georgia in 1974 (Weaver, 1974). The gum exudation on trunks, scaffold limbs, and branches significantly supresses tree growth and fruit yield of susceptible peach varieties. It is one of the most destructive peach diseases in the south of China (Fan et al., 2011) and the southeastern United States (Weaver, 1974; Britton and Hendrix, 1982). Based on the conidial morphology, cultural characteristics, and nucleotide sequences, three *Botryosphaeria* fungi species were reported to be the main pathogens causing the peach gummosis disease: *Botryosphaeria dothidea* (anamorph *Fusicoccumaesculi*), *Botryosphaeria rhodina* (anamorph *Lasiodiplodia theobromae*), and *Botryosphaeria obtuse* (anamorph *Diplodiaseriata*) (Weaver, 1974). Of these, *Botryosphaeria dothidea* is the most common cause of the disease in a large number of hosts worldwide (Britton and Hendrix, 1982; Mancero-Castillo et al., 2018), while *Lasiodiplodia theobromae* has proven to be the most virulent, causing the largest lesions and most copious gummosis in China (Fan et al., 2011).

Previous studies on controlling peach gummosis disease have mainly involved chemical and biological controls with very limited efficacy. Therefore, the use and the breeding of gummosis resistance cultivars are the most cost-effective, environment-friendly, and healthy approach for long-term management of the disease (Beckman et al., 2011). As previously reported, although most peaches and nectarines are susceptible to gummosis disease to some degree, highly resistant genotypes also exist (Beckman and Reilly, 2005). However, using these resistant genotypes in breeding programs *via* conventional breeding methods remains a challenge due to the large plant size, self-compatibility, low genetic diversity, and the most restrictive factors, including the long juvenile periods and breeding cycles (Li et al., 2013; Aranzana et al., 2019). In addition, phenotypic variation of peach gummosis is always affected by several factors, such as wounding, pathogen infection, temperature, or humidity. As far as the genetic factor is concerned, a dominant allele for peach fungus gummosis resistance has been found in almond based on F_1_ and BC_1_F_1_ population. Segregation and mapping analyses located the peach fungal gummosis resistance locus on chimeric linkage groups 6–8 near the leaf color locus (Mancero-Castillo et al., 2018). Furthermore, being the center of origin of peach, China has a huge population of wild relatives and landraces with high genetic diversity (Li et al., 2013; Micheletti et al., 2015). These genetic resources always exhibit specific phenotypes of resistance and fruit quality but are rarely used in modern peach breeding programs. For example, *P. davidiana* carries resistance genes against the peach green aphid and can be used for aphid-resistant breeding (Li et al., 2019). It will be, therefore, helpful to perform extensive work with large-scale germplasm to elucidate the genetic mechanism controlling the severity of peach gummosis disease.

Next-generation sequencing technologies have not only promoted the development of genetics and genomics tools, but also greatly improved the understanding of the genetic basis of important agronomic traits. Based on RNA-seq technology, a large number of differentially expressed genes have been identified, which has enabled the elucidation of the molecular mechanism of plant-pathogen interaction of peach fungal gummosis after the infection (Gao et al., 2016). These genes have been found to be mainly involved in the process of cellular defense and metabolism of carbohydrates, the phenylpropanoid biosynthesis and metabolism pathway, anthocyanin biosynthetic pathway, and the ethylene and jasmonic biosynthetic pathways (Gao et al., 2016). Recently, the reactive oxygen species (ROS) production-scavenging system has been reported to play a crucial role in plant-pathogen interaction and in the development of gummosis caused by *Lasiodiplodia theobromae* (Zhang et al., 2020).

Genotyping by sequencing (GBS) is a method that combines the enzyme-based complexity reduction and the second-generation sequencing technology for marker discovery, with and without the reference genomes (Elshire et al., 2011). Despite the high rate of missing values in the GBS data, the advantages of simultaneous discovery of abundant single nucleotide polymorphisms (SNPs) at low cost, reduced the ascertainment bias compared with array-based markers, and a relatively easy automation still make it an efficient approach to detect polymorphisms and to identify various loci controlling traits both by biparental quantitative trait locus (QTL) mapping and by genome-wide association study (GWAS) (Elshire et al., 2011; Poland and Rife, 2012; Jarquín et al., 2014; Arruda et al., 2016; Minamikawa et al., 2018). A large number of studies on GWAS that were integrated with GBS have been reported in multiple plant species (Arruda et al., 2016; Cao et al., 2016, 2019; Guo et al., 2019; Siddique et al., 2019). By combing GBS-based QTL mapping with GWAS, 117 significant SNPs across the genome were identified to be associated with *P. capsici* root rot resistance in pepper (Siddique et al., 2019). Similarly, the genetic determinants of grape berry-related traits, including grape skin color, berry development period, berry weight, berry flesh texture, and berry flavor, were identified by performing GWAS with 179 grape accessions and 32,311 SNP markers derived from GBS analysis (Guo et al., 2019). Another example of GBS-based GWAS is where the candidate genes of 12 agronomic traits and selected domestication traits, including fruit shape, fruit color, fruit hairy, fruit weight, sorbitol, and catechin content, have been identified (Cao et al., 2016, 2019). Thus, keeping the above in view, an integrated approach of GWAS and comparative transcriptome was used in the present study. Here, the gummosis disease was scored in the large-scale peach core germplasm accessions, grown in the experimental field over the period of 2 years. The plant resources were selected from the previous genetic diversity study (Li et al., 2013). A group of highly resistant accessions, especially the traditional landraces, were identified. These are potentially resistant parents to enrich the gene pool in modern peach breeding programs. The GWAS, combined with RNA-seq, was used to identify the associated SNP markers and candidate genes. The aim was to gain insights into the genetic basis of this complex trait and to apply the results in a peach genomic selection breeding program.

## Materials and Methods

### Plant Materials and Growth Conditions

A set of 195 peach accessions originating from 19 provinces and autonomous regions in China and United States, Italy, New Zealand, and Japan was selected (Supplementary Table 1). All trees were grafted on “MaoTao” rootstock and planted in the peach experimental trial fields of Shanghai Academy of Agricultural Sciences, Shanghai (N30^°^55′3.18″-E121^°^27′14.44″) during the March month of the year 2016. This region in China is characterized by high temperature and high humidity as the annual average temperature and humidity reach up to 17^°^C and 80%, respectively. The tree plants were managed under uniform conditions of irrigation, fertilization, and pest and disease control. Two accessions “Nan Shan Tian Tao” and “Sunfre” were additionally grown in two different locations as replications for resistance validation.

### Evaluation of Lesions and Statistical Analysis of Gummosis Disease Score

The severity of peach gummosis was investigated in the end of the years 2018 and 2019. The score (0, 1, 3, 5, 7, and 9) for each tree was based on the number and area of gumming lesions on the trunks and limbs, a standard evaluation criterion adopted by the modern Chinese peach industry technology community. The minimum score of 0 refers to no visible symptoms or lesions on the whole tree, and the maximum 9 indicates a very severe infection on limbs and the main trunk. The scoring was as follows: 1, only 1–2 lesions with a diameter of the spots less than 3 cm identified on trunks or main limbs; 3, the lesion was from 1 or 2 spots covering an area of up to 25% of the whole plant with the gum spots not clearly distinguishable; 5, the total lesion area was 25–50% of the whole plant with the gum spots not clearly distinguishable; 7, the total lesion area covered from 50 to 75% of the whole plant with the gum spots not clearly distinguishable; and 9 when the total lesion area was more than 75% of the whole plant (Supplementary Figure 1). Those with mean scores of both years as stable to 1 or less were designated as high resistant, and those scores lower than or equal to 3 as middle resistant, and those greater than 3 were designated as susceptible.

The severity of disease was also compared in different peach groups (Supplementary Table 1) divided according to geographic origin and four phenotypes, including fruit pubescence (peach/nectarine), fruit shape (round/flat), fruit flesh color (red/yellow/white), and blossoming time (very early/early/middle/late). The statistical analyses, including means, standard errors (SE), and the minimum and maximum values, were calculated using Graphpad Prism 8 software (Graphpad Software Inc., San Diego, California). Pearson correlation coefficients between the severity score of gummosis disease and geographic origin and four phenotypes were analyzed with the same software. The statistical significance was set at the *p* < 0.001 level. Ordinary one-way ANOVA and unpaired *t*-test (for fruit shape and hairy fruit skin) were used for paired and multiple comparisons, respectively.

### Estimation of Best Linear Unbiased Prediction Values

Best linear unbiased prediction (BLUP) values were extracted from the 2-year (2018–2019) phenotypic data for gummosis disease using the linear mixed model in R-package lme4 based on the following equation:


$$Y_{ij}=\mu +A_{i}+y_{j}+e$$

where Yij is the vector of severity observation for each accession in each year, μ is the overall mean values for all individuals, Ai is the random effect of the ith individual accession (i = 1,……, 195), y_*j*_ is the random effect of the jth year (j = 2018 and 2019), and e is the residual error. Extraction of the random effects (accessions) in the model used the “ranef” function. The estimated BLUP values were used as phenotype values in the GWAS. In the equation, the ratio of the individuals’ (accessions’) variance in the total variance was used as estimated heritability (general heritability). The total variance was the sum variance of accessions, years, and e, that is the same as observations variance.

### DNA Extraction, Re-sequencing, and Single Nucleotide Polymorphism Discovery

Young leaves with no disease from each accession were collected and frozen at -80°C. Total genomic DNA was isolated from 0.1-g tissue using the DNeasy 96 Plant Mini Kit (Qiagen, CA, United States) following the manufacturer’s protocol. The libraries with an insert size of 500 bp were constructed and sequenced by Novogene Bioinformatics Technology Co., Ltd. (Beijing, China) using an Illumina HiSeq X Ten platform (Illumina, San Diego, CA) based on a paired-end mode, which resulted in sequenced fragments of 150 bp read length. The sequencing depth of each accession was greater than 10.33-fold with an average genome coverage of 98.14%. The raw sequencing data and SNP calling were analyzed using SAMTOOLS software (Li et al., 2009). The SNPs were filtered under the quality control parameter to remove those with more than a 7% individual missing rate and a minor allele frequency (MAF) that is lower than 0.05, according to the user manual of Beagle software 3.3.2.

### Estimation of Population Structure, Genetics Parameters, and Genome-Wide Linkage Disequilibrium

The narrow sense of heritability of gummosis disease was estimated by GAPIT3 based on a mixed linear model using whole of the marker data. Principal Component Analysis (PCA) and Neighbor Joining (NJ)-tree analysis were performed to find the clustered group and the genetic distance using GAPIT3 software (Wang and Zhang, 2021) for understanding the population structure. Eigen values and matrices were extracted as dimensionality reduction vectors from all genotype information. The first two PCs with major genetic variance were used to indicate population stratification. The clustered kinship was used to plot the NJ tree. To estimate the rate of linkage disequilibrium decay, *r*^2^ values between each loci genotype were calculated using PopLD decay, which is a fast and effective tool for linkage disequilibrium decay analysis based on variant call format files (Zhang C. et al., 2019). A window size, with averaged 300 kb across the whole genome, was used to calculate average *r*^2^ values.

The genetic diversity indices for different populations, including observed heterozygosity (Ho), inbreeding coefficient (*Fis*), nucleotide diversity (π), and hapotypes, were calculated using the POPULATION program in the stacks package with a custom Perl script. Paired F-statistics values (*Fst*) (Weir and Cockerham, 1984) were calculated to measure the difference between populations using the same aforementioned program. Analysis of molecular variance (AMOVA) was used to partition the genetic variation into inter- and intra- gene pool diversities using Arlequin version 3.5.1 with 1,000,000 markov chain and 100,000 burning steps (Excoffier and Lischer, 2010).

### Genome-Wide Association Study

Gummosis disease severity data from 195 peach accessions were used for GWAS based on mixed linear model (MLM) (Segura et al., 2012), fixed and random model circulating probability unification (FarmCPU) (Liu et al., 2016), and Bayesian-information and linkage-disequilibrium iteratively nested keyway (BLINK) (Huang et al., 2019) using GAPIT 3 in R (Wang and Zhang, 2021). The first three principal components were used as covariates for the population structure and familial relatedness calculation, while the kinship matrix was used to eliminate GWAS false positive. The individual relationships were estimated by using the VanRaden method in the GAPIT3 software (VanRaden, 2008). In each step, the variances were estimated by generalized least-square (GLS), and the *P*-values estimated using the *F*-test. All R scripts for converting data format, estimating phenotype BLUP, and plotting pairwise correlation of LD were coded by our research group, and GWAS programs were performed with default parameters in the GAPIT3 software (Wang and Zhang, 2021). The estimated BLUP values were used as phenotype values in GWAS with the cutoff threshold set as 0.01 and the Bonferroni correction (0.01/total number of markers) to filter the significant markers.

### Estimation of Linkage Disequilibrium Block in the Gummosis Disease-Associated Region and Candidate Genes Identification and Their Annotation

A 100-kb region flanking the significant SNPs associated with gummosis disease and located within the high LD regions was investigated based on the peach genome v2.0 to identify the annotated genes. The annotated gene sequences of the peach genome v2.0 assembly were retrieved from GDR^1^ to identify the target genes for the corresponding associated regions. Pair-wise LD between markers was calculated as the squared correlation coefficient (*r*^2^) of alleles using the R package LD heatmap (Shin et al., 2006). We used *r*^2^ > 0.6 to filter the candidate regions.

### RNA-Seq of the Branch Tissue After Pathogen Inoculation

The 1-year-old branches of the susceptible cultivar “Huyou018” were inoculated with *Botryosphaeria dothidea*. The pathogen was isolated from our own germplasm. The inoculation method was based on a previous study by Gao et al. (2016). The tissue measuring 0.5 cm in a diameter was cut from the lesion area and frozen at -80° C for RNA extraction at 0, 48, 60, 72, and 84 h after inoculation. Total RNA extraction and first-strand complementary DNA (cDNA) synthesis were carried out according to Li’s method (Li X.W. et al., 2015). The sequencing libraries were generated using the NEBNext^®^ Ultra™ RNA Library Prep Kit for Illumina^®^ (NEB, United States) following the manufacturer’s recommendations. Reference genome and gene model annotation files were downloaded from the genome website.^2^ The mapped reads of each sample were assembled by StringTie (v1.3.3b) using a reference-based approach (Pertea et al., 2015). A quantification of gene expression level feature Counts v1.5.0-p3 was used to count the read numbers mapped to each gene. The FPKM of each gene was then calculated based on the length of the gene and reads count mapped to that gene. DESeq2 R package (Love et al., 2014) was used for differential gene expression analysis of pair-wise stages using a model with the negative binomial distribution. The *P*-values were adjusted using the Benjamini and Hochberg’s approach to controlling the false discovery rate. Genes with an adjusted *P*-value < 0.05 found by DESeq2 were assigned as differentially expressed. For each sampling stage, three biological replicates were combined for further DEG analysis.

## Results

### Phenotypic Evaluation of Peach Gummosis Disease and Its Heritability

**FIGURE 1:**
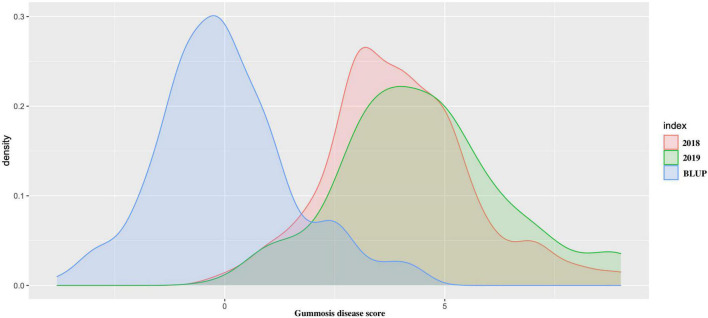
The density distribution of peach gummosis in 195 peach accessions. Disease scale of 0–9 was used, where 0 and 1 represent highly resistant and 9, highly susceptible accessions. The red density distribution is the phenotypic data obtained from 2018; green is the density distribution of the phenotypic data obtained from 2019, and blue represents the density distribution of the best linear unbiased predictions (BLUPs) expressed as estimated breeding values.

The average gummosis disease value score from the 2-year dataset displayed continuous normal distribution ranging from 0 to 9. The average disease score value for each accession was highly consistent across the period of 2 years (*r*^2^ = 0.726). The estimated BLUP value also had a normal distribution (Figure 1). The results demonstrated the existence of a group of accessions highly resistant to gummosis disease. In 92 (47.6%) accessions, the severity of the disease increased over time from 2018 to 2019. In nine accessions, only one to two lesions were found on the entire trunk and branches with a gummosis disease score value of 1, indicating high resistance. Two highly resistant accessions “Nan Shan Tian Tao” and “Sunfre” were grown and validated in two field locations. In 2019, 52 accessions had a gummosis disease score under or equal to 3. A total of 134 accessions had a score greater than 3. Of these 31 accessions, including the well-known traditional landraces from several geographic locations, especially north China (“Shenzhou Bai Tao,” “Feicheng Hong Li 6,” “Feicheng Bai Li 10,” and “Taiyuan Shui Mi”) had very severe disease symptoms ranging from 7 to 9.

**FIGURE 2:**
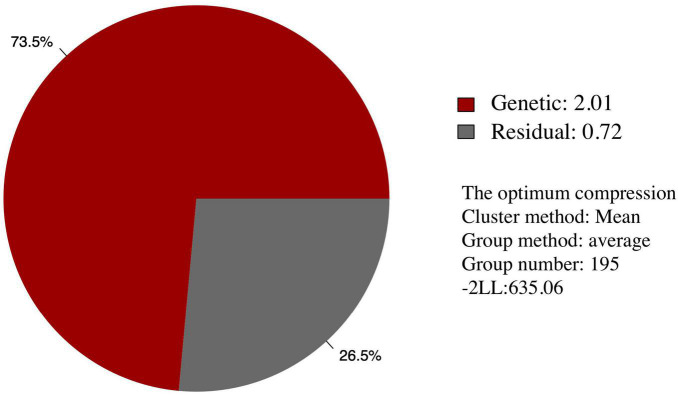
Narrow-sense heritability of the resistance of peach gummosis disease calculated by whole genome SNP markers. “Genetic” and “Residual” means estimated genetic and residual variance in the mixed linear model. The optimum compression information indicates the optimal algorithm to calculate the group kinship matrix, the optimal clustering algorithm, the optimal number of groups in the compress mixed linear model. “-2 LL” is the abbreviation of -2 multiply likelihood value, which means the level of model fitting.

Among the different peach groups, no significant correlation was observed between gummosis disease and the hairy fruit skin (*r*^2^ = 0.0004), fruit flesh color (*r*^2^ = 0.0004), fruit shape (*r*^2^ = 0.002), geographic origin and domestication history (*r*^2^ = 0.0006), and blossoming date (*r*^2^ = 0.001). However, the disease severity score in different peach groups separated by geographic origin and domestication history was significantly different. The mean disease severity score of landraces from South China was lower than that of improved accessions from South China and landraces from North China. The mean score in the nectarine group was relatively higher than that in the peach group (Supplementary Figure 2). The broad-sense heritability estimated by multiple years phenotypic data was approximately 70% (Table 1). The narrow-sense heritability estimated by whole genome DNA markers was 73% (Figure 2).

**TABLE 1:** Variance components, standard deviations of the variance components, and broad-sense heritability of peach gummosis disease evaluated over 2 years in 195 peach accessions.

	Accessions	Years	Residuals	Total
Standard deviation	1.72589	0.5221	0.99049	–
Variance	2.9787	0.2726	0.9811	4.2324
Number of observations	195	3	–	1,389
Heritability	0.70			

### Single Nucleotide Polymorphism Discovery

A total of 1.35 TB of sequence data was generated for the 195 peach genotypes, including 864.45 million reads. The sequencing coverage of at least 1 X was 79.46%. The Q30 ratio, Q20 ratio, and GC content were 85.19, 93.78, and 37.51%, respectively. High-quality reads were aligned with the *Prunus persica* Whole Genome Assembly v2.0 & Annotation v2.1.^3^ A total of 9,486,722 SNPs were initially obtained for these genotypes from the SAMTOOLs utility calling (Li et al., 2009). After removing those SNPs with a MAF lower than 0.05 and the missing value higher than 0.07, the remaining set of 145,608 high-quality SNPs was used for further analysis. Among the 145,608 SNPs, 145,456 SNPs (99%) covered all eight chromosomes. The largest number of high-quality SNPs was found on Chromosome 1 (30,358 SNPs), followed by Chromosome 6 (20,173 SNPs); whereas, the smallest number of SNPs was found on Chromosome 8 (13,718 SNPs). The distribution of SNPs on each chromosome was largely consistent with the physical length of the corresponding chromosome.

### Population Structure, Genetic Diversity, and Linkage Disequilibrium

The observed heterozygosity per individual ranged from 0.068 to 0.332 with a mean of 0.19 (Supplementary Table 2). The highest value was observed for accession “Hu Zhen 43,” while the lowest value was observed for the traditional *Prunus. Ferganensis*, “Mo Yu 8.” The average value of observed heterozygosity of all SNPs was 0.25. The highest value was observed on Chromosome 4 (0.23), and the lowest value was observed on Chromosome 5 (0.15) (Supplementary Table 4 and Supplementary Figure 3).

**FIGURE 3:**
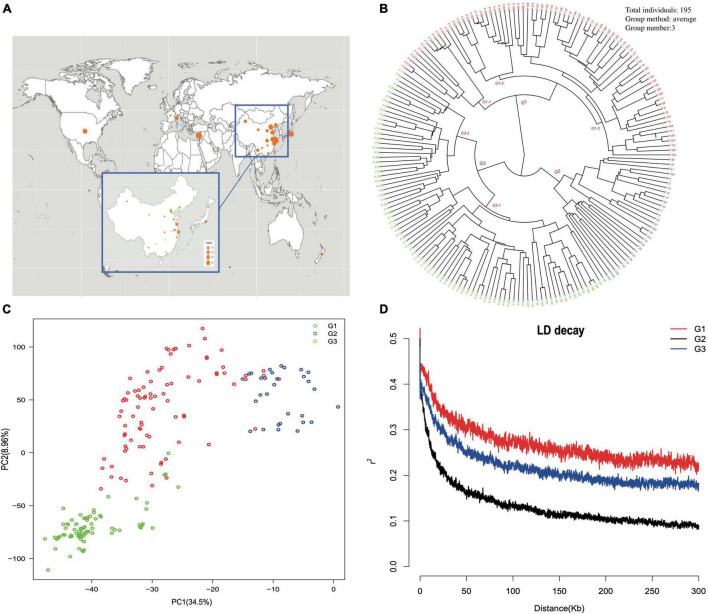
**(A)** The geographic location of the origins of each accession in China and worldwide. **(B)** Phylogenetic dendrogram constructed by the neighbor-joining method. The accession name is represented by the accession code, which is coincident with Supplementary Table 1. **(C)** Principal component analysis (PCA) of accessions, with the proportion of variance explained by each PC indicated in parenthesis. Dots of different color indicate different cluster groups. **(D)** Linkage disequilibrium measures (*r*^2^) against physical distance between pairs of SNP markers for the three major groups.

The geographical origin of the selected accessions could be located at three different continents and 19 provinces in China (Figure 3A). A phylogenetic dendrogram using the neighbor-joining method clustered 195 accessions into three major groups, mainly according to fruit shape, hairy vs. glabrous fruit skin, pedigree, geographical origin, and domestication history (Figure 3B, Supplementary Table 1, and Supplementary Figure 4). The first major group was composed of 79 accessions and further divided into three subgroups. The first subgroup G1-1 was marked as the flat peach group, with 10/12 accessions being flat peaches. The accessions of the other two subgroups were closely related to the founder “Shanghai Shui Mi” used in peach breeding programs of China and Japan. One of the most famous cultivars, “Yu Lu,” clustered with the primitive cultivars originating from Shanghai, and most of the Japanese cultivars clustered with “Bai Hua Shui Mi.” The second major group had 30 cultivars and was marked as the traditional landrace group, which included those cultivars carrying special traits, such as red flesh, extremely firm texture, and extremely low chilling requirement. The third major group was composed of 86 accessions and was further divided into two subgroups. The first subgroup G3-1 included 18 peaches and 38 nectarines. In this study, 84% (38/45) of nectarines were clustered in this group. It is noticeable that most of the accessions in this subgroup were characterized as early or very early blossoming. The second subgroup, G3-2, included 27 peaches and three nectarines, and was mainly composed of the accessions with early maturity time. Based on the phylogenetic dendrogram, first approximation of population structure was obtained by using PCA for the complete set of SNPs (Figure 3C). The first two principal components explained 43.46% of the total genotypic diversity. The stratification pattern was highly consistent with NJ hierarchical clustering. All 145,456 SNP markers were employed to estimate the LD extent across the three major groups. The average value of *r*^2^ was 0.269 in G1, 0.133 in G2, and 0.218 in G3. The LD value decreased with distance between the markers in all groups. The level of LD value in G1 was higher than that in G2 and G3. The average value of *r*^2^ dropped below 0.2 at around 30 kb in G2 and 150 kb in G3 (Figure 3D).

Based on the population structure, the genetic variation among three major groups was estimated. The result showed that G2, which was a landrace group, had the highest values of *Fis*, π, and haplotype diversity, while the observed heterogosity value of G2 seemed significantly lower than that of the other groups. The statistical analysis of haplotypes showed that the number of haplotypes and unique haplotypes of G3 was higher than that of G1 and G2 (Table 2). The AMOVA revealed that 12.86% of the total variation was found among groups, while the rest of the variation (87.14%) was within groups (Table 3). The pairwise genetic differentiation (*Fst*) was highest (0.0909) between G1 and G2 and the lowest (0.0494) between G2 and G3.

**TABLE 2:** The genetic diversity estimated of three major group.

Group ID	No. of individual	Obs het	Fis	π	No. of haplotype	No. of unique haplotype	Haplotype diversity
G1	79	0.3653	0	0.2852	13,175,146	161,332	0.2852
G2	30	0.2418	0.2544	0.3256	5,003,220	161,607	0.3255
G3	86	0.3533	0	0.3024	14,342,564	166,600	0.3024

*Obs Het represented observed heterozygosity. Fis indicated inbreeding coefficient. π indicated nucleotide diversity.*

**TABLE 3:** Analysis of molecular variance of the genetic differentiation among and within three major groups of 195 accessions.

Source of variation	d.f.	Sum of squares	Variance components	Percentage of variation	*P*-value
Among groups	2	479,575	1,885	12.86	0.001
Within groups	387	4,944,323	12,776	87.14	0.001
Total	389	5,423,898	14,661		

### Genome-Wide Association Study for Gummosis Disease

**FIGURE 4:**
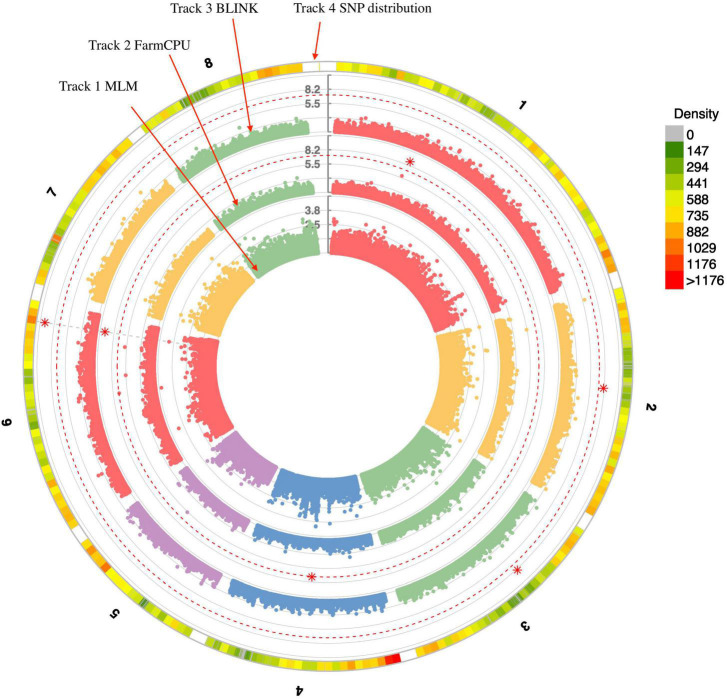
Distribution of SNPs on the eight chromosomes. Tracks 1, 2, and 3 represent results from three statistical analysis models used for genome-wide association study (GWAS) of peach gummosis disease. Track 4 represents filtered SNPs on the eight peach chromosomes. The red dotted-line indicates the significance threshold (-log_10_ *P* = 6). The red asterisks represent the significant SNP.

**FIGURE 5:**
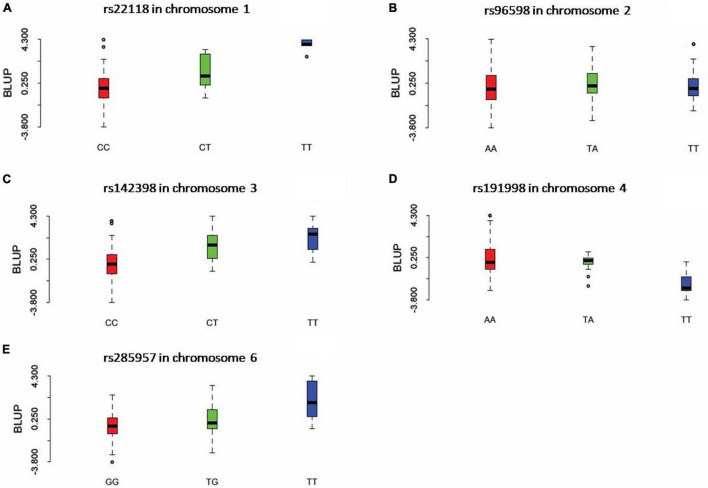
The boxplots show the comparison of the phenotypic performance illustrated by the BLUP value for different genotypes of the five significant SNPs. For each graph, *X* axis indicates the different genotypes of SNP rs22118 **(A)**, rs96598 **(B)**, rs142398 **(C)**, rs191998 **(D)**, and rs285957 **(E)**. *Y* axis indicates the BLUP value.

Three statistical models were used for GWAS to detect the associated genomic regions with gummosis disease using 145,456 SNP markers and the estimated BLUP values. No significant locus was identified by the MLM model. Five SNPs on five chromosomes were identified as significantly associated with peach gummosis disease (Figure 4, Table 4, and Supplementary Figure 5). Three SNPs were detected by FarmCPU and three by BLINK. Among the five SNPs, rs285957 at about 28 Mb on Chromosome 6 was simultaneously detected both by FarmCPU and BLINK methods with the allelic effect of 0.64. The phenotypic variation explained by a single SNP varied from 6.28 to 19.85%, and from 6.98 to 17.94% based on FarmCPU and BLINK, respectively. The variance explained by the SNP rs285975 was different under the two models. There were significant phenotypic differences caused by four SNPs in the different genotypes. The BLUP value of allele “T” at rs22118_C/T, rs142398_C/T, and rs285957_T/G was significantly higher than that of allele “C” and “G,” especially at rs22118. The value of allele “A” at rs191998 was higher than for “T” (Figure 5). It is noticeable that the nine highly resistant accessions carried the same genotype “GG” at rs285957.

**TABLE 4:** A summary of significant SNPs consistently associated with gummosis disease in peach accessions.

Model	SNP ID	Chromosome	Position	*P*-value	MAF	FDR	Allelic effect	Variance explained
FarmCPU	rs22118	1	13,801,843	9.95E-10	0.061538	7.24E-05	1.113248981	19.85
FarmCPU	rs191998	4	12,480,256	5.46E-08	0.092308	0.002648813	-0.732419028	19.03
FarmCPU	rs285957	6	28,139,324	9.15E-11	0.294872	1.33E-05	0.640842547	6.28
BLINK	rs96598	2	13,388,877	7.73E-09	0.464103	0.000375161	NA	6.98
BLINK	rs142398	3	9,810,975	3.20E-09	0.089744	0.000233144	NA	17.94
BLINK	rs285957	6	28,139,324	8.27E-11	0.294872	1.20E-05	NA	17.41

*FDR in the head row refers to “FDR. Adjusted P-values.”*

### Analysis of Differentially Expressed Genes Related to Gummosis Disease at Different Pathogen Inoculation Stages

The fifteen transcriptome sequencing profiles (5 sampling times × 3 replications) generated a total of 124.91 Gb high-quality data with Phred Quality score or Q30 of 93.43%. The total clean reads for each sample ranged from 43.19 to 70.43 million (Supplementary Table 4). The proportion of total mapped reads to the peach reference genome v2.0 accounted for 96.02–97.43%. Of these, the properly mapped reads accounted only 88.31–92.98%. The highest number of DEGs was observed during the first 48 h after inoculation, including the up- and downregulation of 6,451 and 6,592 genes, respectively. The lowest number of DEGs was observed in 72 vs. 84 h after inoculation, including the up- and downregulation of 2,924 and 2,212 genes, respectively.

The functional annotation of DEGs discovered was performed using gene ontology (GO) functional classification and enrichment analyses. The results showed that three biological process (BP) and six molecular function (MF) terms enriched within 48 h after inoculation, including a response to biotic stimulus (GO:0009607). We also identified a considerable number of DEGs from the functional groups of carbohydrate metabolic process (GO:0005975) in “48 vs. 60 h” and “60 vs. 72 h” comparison (Supplementary Table 5). KEGG Pathway enrichment analysis of the DEGs obtained from pairwise comparisons showed that most DEGs were involved in two pathways “Cysteine and methionine metabolism” and “Ribosome” among 0 vs. 48 h. The other three pathways “Flavonoid biosynthesis,” “plant-pathogen interaction,” and “plant hormone signal transduction,” which might be highly correlated with pathogen infection, were also significantly enriched (Supplementary Table 6).

### Linkage Disequilibrium Block in the Gummosis Disease-Associated Genomic Regions and Predicted Candidate Genes

**FIGURE 6:**
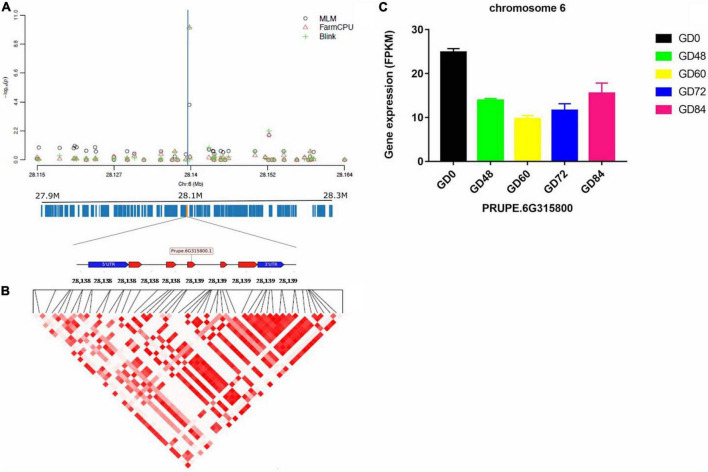
Significant associations and candidate genes on Chromosome 6, underlying peach gummosis disease. **(A)** Manhattan plots showing the significance of SNP rs285957 at the chromosome-wide level. The vertical blue lines indicate the position of the significant locus identified by three different models. The annotated candidate genes and the gene structure are represented below the plot. **(B)** Pairwise correlation of LD (*r*^2^) between significant SNPs along the highlighted genomic region. **(C)** Relative expression of *Prupe.6G315800* obtained by comparative RNA-Seq profile during the inoculation of “Huyou018.”

The linkage disequilibrium (LD) pattern around each identified significant gummosis disease associated SNPs was evaluated by calculating the squared allele-frequency correlation between each pair of these SNPs. The candidate genes for disease resistance/susceptibility were then searched in the genomic regions flanking the associated SNPs. LD analysis revealed a high pairwise correlation among SNPs within two candidate genes (*PRUPE.2G084800* and *PRUPE.6G315800*) on Chromosomes 2 and 6, respectively. The putative gene on Chromosome 2 harboring the significant SNP rs96598 is *PRUPE.2G084800* encoding galactose oxidase. The other gene located at the 2 kb upstream region in the same chromosome is a transcriptional activator *PRUPE.2G084700.* It has shown a higher expression level than *PRUPE.2G084800* (Supplementary Figure 6). Upon pathogen infection, these two genes showed significant upregulation from 0 to 72 h and then were downregulated. The significant SNP rs285957 located on Chromosome 6 was found in *PRUPE.6G315800* encoding a Dna J domain, which plays an important role in plant biotic stress in *Arabidopsis* (Wang et al., 2014). The transcript of *PRUPE.6G315800* decreased after pathogen infection and then increased from 60 to 84 h (Figure 6). The third associated SNP rs142398 located on Chromosome 3 was found within the coding region of a leucine-rich repeat receptor-like protein kinase (LRR-RLK) *PRUPE.3G116000*. Pathogen infection dramatically downregulated the expression level of *PRUPE.3G116000* from 0 to 48 h. The log_2_Fold Change of the transcript level of *PRUPE.3G116000* was reduced 3.96 times at 48 h after inoculation compared to 0 h (Supplementary Figure 7). The associated SNP rs191998 on Chromosome 4 was located within the putative gene *PRUPE.4G201700*, which showed an expression pattern similar to *PRUPE.3G116000*. The functional annotation of *PRUPE.4G201700* identified it as a histone H2A.1-like protein (Supplementary Figure 8). Additionally, two UDP-glucosyl transferase genes (*UGTs, PRUPE.1G169100*, and *PRUPE.1G169200*) were found to be located at the 20 and 31 kb region downstream of the significant SNP rs22118 on Chromosome 1. However, the pairwise correlation was extremely low within the 25 kb region around the significant SNP (Supplementary Figure 9). The expression of the two genes significantly increased on pathogen inoculation until 72 h, which were similar to *PRUPE.2G084700* and *PRUPE.2G084800*.

## Discussion

Gummosis disease is one of the most destructive diseases causing severe loss to peach industry worldwide. At present, no orchard management or fungicide effectively controls the disease in the peach field (Beckman et al., 2011). The absence of resistance cultivars to be used as parents and the poorly understood genetic mechanisms of gummosis disease are the major challenges for breeding the resistant cultivars. In addition, the best solution for breeding a resistant cultivar should be coupled with high fruit quality and not just the root stock, as, even if the rootstock is resistant, the cultivars grafted on the rootstock remain susceptible. In the present study, we investigated large-scale germplasm resources, including improved cultivars and traditional landraces, to identify resistant cultivars for parental selection for future breeding programs. Integrating multiple approaches, including multi-model GWAS and RNA-seq, five novel genetic loci associated with gummosis disease were identified. Four genetic loci with SNPs located on Chromosomes 1, 3, 6, and 4 were found to have a significant impact (*P*-value < Bonferroni threshold) on the disease severity. The candidate genes harboring the significant SNPs (rs142398, rs285957, and rs191998) were mainly identified and validated by comparative RNA-seq. The functional annotation showed that these genes were highly related to disease response, which further indicates the reliability of our results.

### Phenotypic Variation and the Selection of Resistant Sources

Gummosis is a complex disease as it is reported to be affected by multiple external factors, such as age of the tree, pruning methods, orchard management, temperature humidity, as well as the pathogenic type, plant regulators, and the cultivar itself. By comparing the disease severity of plants in the years of 2018 and 2019, we confirmed the results of Mancero-Castillo that the severity of gumming increases with the age of the tree (Mancero-Castillo et al., 2018). Then, to explain the proportion of phenotypic variance determined by genetic factors, we firstly evaluated the heritability of gummosis disease. The high heritability estimated for peach gummosis disease based on multi-years phenotypic data and genome-wide SNP data is strong evidence that the phenotypic variation of the disease is largely due to genetic effect. This, moreover, indicated the possibility of breeding for resistant cultivar to gummosis disease by introducing resistant parents in the breeding program. Also, because of the high heritability and the significant correlation reported among gum exudates, disease severity, and tissue necrosis (Mancero-Castillo et al., 2018), we mainly evaluated the severity of gummosis disease according to the area of the wound lesion on trunks and branches in the same field, but not according to the pathogen inoculation. Our phenotypic results of 195 germplasm sources showed that most accessions were moderately or highly susceptible to gummosis. Nine *Prunus persica* accessions were highly resistant, of which two accessions showed consistency in resistance across different growing conditions. This result is congruent with the previous reports, where the majority of the peach genotypes were susceptible and no sources of complete resistance were identified even though the available peach genetic resources show the presence of resistant genotypes upon investigation in the field after pathogen infection (Beckman and Reilly, 2005; Beckman et al., 2011). One of the resistant cultivars, “Nan Shan Tian Tao,” is a landrace originating from Shenzhen, southern China, a place with high humidity and temperature (the annual average temperature and humidity ranges up to 25 and 68.5%). This cultivar also exhibited high gummosis resistance in Jiaxing, Zhejiang. The resistance of “Sunfre” was validated by growing in two different locations with “3 plus 6” replications in total. Our previous research on 8-year-old trees of “Sunfre,” grown in different experimental fields, also exhibited high resistance to gummosis disease with strong tree vigor and smooth clean trunk (Supplementary Figure 10). The consistent and stable resistance of these two cultivars made them more reliable selections as breeding materials. Although a genetic source for resistance to peach fungal gummosis has been reported from the *P. dulcis* cultivar “Tardy Nonpareil,” which is an almond cultivar (Mancero-Castillo et al., 2018), but it may be simpler and more efficient to choose some famous landraces of *Prunus persica* for intraspecific cross so as to introduce specific alleles, increasing the genetic diversity, and selecting resistant progenies. Accessions with low disease severity will not only be the ideal materials for breeding superior resistant cultivars but also for identifying disease resistance and related genes in future genetic studies.

Several research groups have reported gummosis disease severity in various peach cultivars. However, there are less comparative studies in different peach groups. Zhao et al. (1996) reported that the severity of the disease was lowest in nectarine groups and highest in the flat peach group. Although we did not identify significant differences on gummosis severity in the groups based on hairy fruit skin, blossom time, and fruit flesh color, a major difference was observed in the groups divided by geographic origin. For example the lowest gummosis disease score was observed in the South China landrace group, and the highest gummosis disease score was observed in the North China landrace group. This result was further confirmed by comparing the disease severity among different subgroups clustered using the neighbor-joining method with genome-wide SNP markers (Supplementary Figure 2). Subgroup G1-2, which contains most of the primitive landraces and offsprings originating from Shanghai, was more resistant than the other subgroups. Shanghai is in Southeast China and has high temperatures and humidity during summer. The monthly average temperature and humidity from June to September range from 23.3 to 28.9^°^C, and 8 to 85%, respectively. It is known as the origin of flavorful honey peach and elite commercial cultivars worldwide. The result that peach genotypes originating from the Shanghai region showed high resistance to gummosis disease, therefore, indicates toward a selective adaption to climate during acclimation or evolutionary history. The pedigree of modern cultivars is another reason for resistant gene inheritance. For instance, the symptoms in “Early Red 2,” an offspring of “Sunfre” were mild compared to “Huyou” nectarines, derived from “Mayfire,” which had severe disease symptoms (Supplementary Table 7).

### Population Structure and Genetic Diversity

Population structure obtained from Neighbor-Joining algorithm and PCA was highly consistent and has clustered the accessions according to the domestication history, pedigree, geographic origin, fruit shape, fruit hairy skin, and blossome time. The traditional landraces were clearly separated from the improved accessions, which, as per the previous reports, were obtained by SSR markers and by the GBS-based SNP array (Li et al., 2013; Micheletti et al., 2015; Cao et al., 2019). Because the accessions originating from Shanghai or developed from “Shanghai Shui Mi” are derived from the same parents, they were grouped together in G1. It is remarkable that the two traditional honey peach (“Yu Lu” and “Bai Hua Shui Mi”), which might have been introduced from Shanghai showed close clustering with the primitive landraces from Shanghai. Henceforth, they are not only the ancestors for the elite cultivars in modern peach breeding programs, but also the most popular cultivars in the fresh-eating market due to the favorable aroma, juicy, melting texture, and high sweetness.

The genetic diversity indices provided useful information on genetic diversity of each population. The high level of genetic diversity within groups and a low level of diversity among groups may be due to gene flow and artificial selection (Eltaher et al., 2018). The low observed heterozygosity and the highest value of *Fis*, π, and haplotype diversity among landraces of G2 can be strong indications that there are no gene flow from landrace in the current breeding program. However, the higher observed heterozygosity and lower genetic diversity among accessions of G1 and G3 may be due to artificial selection of favorable morphological traits and narrow genetic bottleneck because most accessions in these two groups were improved cultivars with desirable traits, such as glabous fruit skin, strong aroma, sweetness, and low acidity. Some accessions have been frequently used as crossing materials (Li et al., 2019). Considering the above results, the understanding of genetic and phenotypic diversity of G2 will be very helpful for introducing new alleles and enlarging the genetic diversity for creative cultivar selection in the future.

### Genome-Wide Association Study Model Selection and Quantitative Trait Locus Identification

Optimal statistical models are needed to accurately evaluate the associations between markers and phenotypes. On comparing the results of three statistical models, no significant SNPs were detected by MLM, which is known as a single locus marker testing model for association study. Many studies have shown that multiple loci markers testing models, such as FarmCPU and BLINK, are more powerful for detection of real association signals and have been integrated into GAPIT3 R packages (Wang and Zhang, 2021). These utilize different testing models to select pseudo QTNs as fixed effect in the final estimated model. FarmCPU uses a set of markers associated with a causal gene as a cofactor instead of kinship to avoid overfitting and eliminates confusion between kinship and testing markers iteratively (Liu et al., 2016). The BLINK eliminates the requirement of FarmCPU, which demands that the quantitative trait nucleotides (QTNs) should be evenly distributed in the genome (Huang et al., 2019). The simulation study has shown that the BLINK model is more powerful than the FarmCPU (Wang and Zhang, 2021). In this study, we compared the results from these two multiple loci models (FarmCPU and BLINK) and selected all significant markers from both models as candidate loci for gummosis disease resistance.

Remarkably, at present, there is only one publication on QTL mapping of peach gummosis disease, which identified a locus Botd8 on chimeric linkage groups 6–8 from “UF Sharp” × (FG × TNP1260), with the effect on gumming rates ranging from an average of 0.5 ± 0.2 for resistant to 3.4 ± 0.2 for the susceptible trees (Mancero-Castillo et al., 2018). Here, we identified a total of five quantitative resistance loci affecting gummosis disease by multiple GWAS resolution. All five significant SNPs-harboring genomic loci distributed on five chromosomes (1, 2, 3, 4, and 6) are novel and provided high variance explanation. The large allele effect on phenotypic value is a good indication for detecting the favorable resistance alleles in the current population as well as for future populations. The higher number of QTLs identified by GWAS might be due to higher genetic diversity of our germplasm since most accessions were selected based on the previous study of 658 oriental and occidental cultivars (Li et al., 2013). In addition, comparing with previous linkage mapping study using bi-parental populations, GWAS gave high mapping resolution to narrow down the chromosomal region of candidate QTLs and predict causal genes (Zhang et al., 2016). However, the SNPs found in our study have not yet been validated in multiple bi-parental populations or natural populations, especially elite parents. This means that there is need for validating the SNPs either using KASP or other convenient and effective tool in training populations to identify favorable alleles that can be selected in future marker-assisted parent selection (MAPS) or marker-assisted seedling selection (MASS) breeding programs.

### Identification of Gummosis Disease Resistance Loci and Candidate Genes

Several studies have reported that peach has large LD extent, spanning from around 25–50 kb due to its self-compatibility with limited genetic diversity to be used in peach breeding (Li et al., 2013; Micheletti et al., 2015; Cao et al., 2016). In our study, the LD extent seems to be highly dependent on different groups as it ranged from 30 kb (in G2) to 150 kb in G3. This study is similar to the previous reports (Li et al., 2013; Micheletti et al., 2015; Thurow et al., 2020). However, the LD extent detected for G1 was relatively larger than for G2 and G3. This may be because most accessions in this group have originated from Shanghai or derived from “Shanghai ShuiMi.” With the above view in mind, candidate genes within a conservative window size of approximately 100 kb were searched, and their LD level was analyzed. The SNP rs285927 located in a Dna J domain was identified using both FarmCPU and BLINK models. This is a protein, also known as heat-shock protein 40, which belongs to the family of conserved co-chaperones for HSP70s. Plant J-domain proteins have been shown to have diverse functions in stress responses. For example, silencing a soybean type-III nuclear body-localized DnaJ protein GmHSP40.1 enhanced the susceptibility of soybean plants to soybean mosaic virus (Liu and Whitham, 2013). Similarly, the overexpression of tomato chloroplast-targeted DnaJ protein (LeCDJ2) enhanced the tolerance to drought stress and resistance to *Pseudomonas solanacearum* in transgenic tobacco (Wang et al., 2014). However, virulence effector HopI 1, a chloroplast-targeted class-III J protein from *Pseudomonas syringae*, has been shown to suppress both salicylic acid accumulation and host defense responses in *Arabidopsis* (Jelenska et al., 2007). The comparative transcriptome analysis in this study identified 39 differentially expressed genes that were annotated as Dna J domains. Of these, *PRUPE.6G315800* was co-localized at the same region, where significant SNP rs285957 was detected by GWAS. Moreover, its transcript level also decreased significantly after pathogen inoculation. To further ascertain the function of the DnaJ domain gene family in peach, genome-wide identification and characterization combined with transcript analysis and subcellular localization are necessary. Additionally, another putative gene, *PRUPE.6G315700*, encoding the calmodulin-binding-like protein (CBP), which is related to disease resistance against *Pseudomonas syringae* in *Arabidopsis* and tomato, was found at the 7 kb upsteam of the significant SNP locus (rs285957) (Chiasson et al., 2005). In peach, it has been reported that exogenous CaCl_2_ treatment can increase the content of Ca^2+^ in shoots, prevent the degradation of cell wall polysaccharides, maintain the stability and integrity of cell wall, and, finally, reduce the severity of gummosis disease (Li M.J. et al., 2015).

The candidate gene *PRUPE.3G116000* harboring the significant SNP rs142398 on Chromosome 3 is the LRR-RLK gene, which belongs to a large gene family of receptor-like protein kinases and actively participates in regulating growth, development, signal transduction, immunity, and stress responses in plants (Liu et al., 2017; Sun et al., 2017). By performing GBS-based bi-parental QTL mapping and GWAS, clusters of candidate nucleotide-binding site-leucine-rich repeat (NBS-LRR) and receptor-like kinase (RLK) were predicted within the QTL region, which was highly associated with *P. capsici root rot* resistance in pepper (Siddique et al., 2019). In peach, 258 *LRR-RLKs* genes have been found (Sun et al., 2017). Here, we identified a total of 11 SNPs within *PRUPE.3G116000* using genome sequencing data. However, the correlation between the significant SNP (rs142398) and the other SNPs in the LD block around the gummosis disease-associated genomic region was lower. For this reason, the targeted region was traced in the peach genome v2 in GDR.^4^ As a result, ten SNPs on the IRSC Peach 9K and 18 K SNP array located in the coding region of *PRUPE.3G116000* were found. It is worth noting that the haplotype block constructed with the peach IRSC 9 K SNP array by Stijn (Vanderzande et al., 2019) was not found in this region. This indicates that it may not be a conserved gene but a highly diverse region resulting from recombination, selection, or domestication. Therefore, use of multi bi-parental populations or BSA is required to further analyze the association of *PRUPE.3G116000* with peach gummosis disease.

The UDP-glycosyl transferases are a multigenic and highly divergent superfamily of enzymes that are widely found in all living organisms. In plants, many UGTs play important roles in plant defense to biotic and abiotic stresses by glycosylating acceptor molecules, such as anthocyanidins, flavanols, flavonoids, saponins, sterols, terpenoids, phenylpropanoids, and plant hormones, or by detoxifying and deactivating xenobiotics as a pivotal role in plant-pathogen interactions. In wheat and barley, several *UGT* genes have been reported to enhance their resistance against *Fusariumhead blight* by glycosylating the deoxynivalenol (DON), produced by *Fusarium* fungus to the less toxic D3G, such as the barley *HvUGT13248 and HvUGT-10W1* (Xing et al., 2016) and the wheat *TaUGT3* (Xing et al., 2016) and *TaUGT6* (He et al., 2020). In peach, 168 *UGT* genes have been identified and clustered into 16 groups based on the phylogenetic analysis (Wu et al., 2017). Using the RNA-seq technique, six *UGTs* (*ppa005290 mg*, *ppa023599 mg*, *ppa012496 mg*, *ppa005161 mg*, *ppa025073 mg*, and *ppa016033 mg*), which are mainly involved in the biosynthesis of anthocyanidins and other flavonoids, has been shown to be upregulated by pathogen infection. The tissue around the wounded area changed from green to red and accumulated anthocyanin during disease infection (Gao et al., 2016). It is worth noting that *PRUPE.1G169100* is identical to the *ppa005161* identified in peach genome v1 by Gao et al. (2016). In our study, two *UGTs* located around the significant SNP rs22118 were remarkably upregulated by pathogen infection. They have been previously reported to belong to the same cluster and as homologous with *UGT75D1, UGT84A1*, *UGT74F2*, and *UGT74F2*, playing a critical role in *Pseudomonas syringae* resistance and involved in salicylic acid glycosylation in *Arabidopsis* (Boachon et al., 2013; Thompson et al., 2016). UGT glycosylation is a critical step in forming glycosylated linalool, which has been reported to have a defensive function in several plant species such as against rice bacterial blight induced by *Xanthomonas Oryzae PV. Oryzae* (Xoo) (Antony et al., 2010), citrus canker induced by *Xanthomonas citri* subsp. *citri* (Xcc) (Takehiko et al., 2017) and antibacterial and antifungal activities to Xcc and *Penicillium italicum* in Ponkan mandarin (Shiduku et al., 2013). In peach, *PpUGT85A2* catalyzes the glycosylation of linalool, and the overexpression of this gene increases the production of linalyl-β-d-glucoside (Wu et al., 2019). Here, we did not evaluate the content of anthocyanin or glycosylated linalool in the shoots of different peach cultivars. Although the regulation of anthocyanin biosynthesis and *terpene synthase* genes by UGTs was not investigated, this research provides a new insight into resistance to peach gummosis disease to understand its defense system.

## Conclusion

The present study is the first to identify multiple genetic factors involved in peach gummosis using GWAS by using a substantial number of peach germplasm accessions. Two highly resistant accessions were detected in the germplasm, which will be useful plant material for resistant cultivar selection in peach breeding programs. Strong evidence was provided on its high heritability both by genotypic and phenotypic data for peach gummosis disease. This indicates that the phenotypic variation of this complex trait is largely determined by genetic control. By integrating the GWAS and RNA-seq analysis, four candidate genes harboring the significant SNPs on chromosomes 2, 3, 4, and 6 and showing significant differential expression were identified. This study enhances our knowledge of the genetic basis of resistance to peach gummosis disease. The associated markers and resistant plant sources can assist a precise breeding to develop breeders in developing higher resistant cultivars to the disease at a faster rate.

## Data Availability Statement

The original contributions presented in the study are publicly available. This data can be found here: National Center for Biotechnology Information (NCBI) BioProject database under accession number PRJNA746706.

## Author Contributions

XL and ZY initiated the project, designed the experiments, and selected the core collection. MS, JD, HZ, and XZ collected the DNA samples. LW, WF, and KC provided some peach accessions. XL, JZ, ZG, MZ, JJ, KG, and HJ scored the gummosis disease. XL and JW analyzed the phenotypic and genotypic data. XL drafted the manuscript. XL, JW, ZY, and ZG wrote and reviewed the manuscript. All authors read and approved the final manuscript.

## Conflict of Interest

The authors declare that the research was conducted in the absence of any commercial or financial relationships that could be construed as a potential conflict of interest.

## Publisher’s Note

All claims expressed in this article are solely those of the authors and do not necessarily represent those of their affiliated organizations, or those of the publisher, the editors and the reviewers. Any product that may be evaluated in this article, or claim that may be made by its manufacturer, is not guaranteed or endorsed by the publisher.

## Footnotes

1
www.rosaceae.org

2
http://www.rosaceae.org/species/prunus_persica/genome_v2.0

3
https://www.rosaceae.org/species/prunus_persica/genome_v2.0.a1

4
https://www.rosaceae.org/
