# Supplementary material for: Single Nucleotide Polymorphism Detection for Peach Gummosis Disease Resistance by Genome-Wide Association Study
Source: Front Plant Sci. 2022 Feb 7;12:763618. doi: 10.3389/fpls.2021.763618 (PMC8858797; doi:10.3389/fpls.2021.763618)
Supplement: Supplementary file 1 [file Data_Sheet_1.ZIP › Supplementary figures and tables/Supplementary Material.DOCX]

Supplementary Material

## Supplementary Tables

**Supplementary Table 1** Phenotypic description of the 195 peach genotypes used in this study.

Note: Accession code is the same code with phylogenetic dendrogram (Figure 3B). GDS in the first row indicate the mean value of gummosis disease severity. In RD column, S, M and R means susceptible, middle resistant and high resistant. In “Blossom time” column, VE, E, M and L indicate very early, early, middle and late flowering time. In “Fruit skin hairy” column, N and P indicate nectarine and peach. In “Fruit shape” column, F and R indicate flat and round shape. In “Flesh color” column, R, Y and W indicate red, yellow and white color. In “Geographic origin” column, CS, CN, JA, WT indicate South China, North China, Japan and Western countries. In “Domestication history” column, IV and LR indicate improved cultivars and landrace.

**Supplementary Table 2** The heterozygosity of each individual.

**Supplementary Table 3** The heterozygosity of SNP markers.

**Supplementary Table 4** Statistics of DGE sequencing.

**Supplementary Table 5** Gene Ontology (GO) functional classification analysis of differentially expressed genes based on RNA-Seq data

**Supplementary Table 6** KEGG pathway mapping for differentially expressed genes based on RNA-Seq data.

**Supplementary Table 7** The severity of gummosis disease of the selected resistant/susceptible accessions. GDS in the first row indicate the mean value of gummosis disease severity

## Supplementary Figures

**Supplementary Figure 1** The visualize vision of different gummosis disease severity. Since the complete immune cultivar with the severity of “0” score wasn’t identified in the field, no picture was included for “0” score.

**Supplementary Figure 2** The severity of gummosis disease in different peach groups divided by fruit hairy skin, geographic origin, fruit flesh color, domestication history, fruit shape and blossom date. The abbreviation of X-axis for each graph is consistent with Table S1. The total accession number for blossom date was not 195 due to the missing value of this phenotype.

**Supplementary Figure 3** The frequency distribution of heterozygosity of individuals and SNP markers derived from re-sequencing of 195 accessions.

**Supplementary Figure 4** The zoomed NJ-Tree clearly separated into three major groups.

**Supplementary Figure 5** Manhattan and QQ-plots for peach gummosis disease resulting from the different statistical models, the green horizontal line indicates the significance threshold (−log_10_ P = 6).

**Supplementary Figure 6** Significant associations and candidate genes on chromosome 2 underlying peach gummosis disease. (A) Manhattan plots showing the significance of SNP rs96598 at the chromosome-wide level. The vertical blue lines indicate the position of the significant locus identified by three different models. The annotated candidate genes and the gene structure are represented below the plot. (B) Pairwise correlation of LD (r^2^) between significant SNPs along the highlighted genomic region. (C) Gene expression of *PRUPE.2G084700* and *PRUPE.2G084800* obtained by comparative RNA-Seq profile during the inoculation process of ‘Huyou018’.

**Supplementary Figure 7** Significant associations and candidate genes on chromosome 3 underlying peach gummosis disease. (A) Manhattan plots showing the significance of SNP rs142398 at the chromosome-wide level. The vertical blue lines indicate the position of the significant locus identified by three different models. The annotated candidate genes and the gene structure are represented below the plot. (B) Pairwise correlation of LD (r^2^) between significant SNPs along the highlighted genomic region. (C) Relative expression of *PRUPE.3G116000* obtained by comparative RNA-Seq profile during the inoculation process of ‘Huyou018’.

**Supplementary Figure 8** Significant associations and candidate genes on chromosome 4 underlying peach gummosis disease. (A) Manhattan plots showing the significance of SNP rs191998 at the chromosome-wide level. The vertical blue lines indicate the position of the significant locus identified by three different models. The annotated candidate genes and the gene structure are represented below the plot. (B) Pairwise correlation of LD (r^2^) between significant SNPs along the highlighted genomic region.

**Supplementary Figure 9** Significant associations and candidate genes on chromosome 1 underlying peach gummosis disease. (A) Manhattan plots showing the significance of SNP rs22118 at the chromosome-wide level. The vertical blue lines indicate the position of the significant locus identified by three different models. The annotated candidate genes and the gene structure are represented below the plot. (B) Pairwise correlation of LD (r^2^) between significant SNPs along the highlighted genomic region. (C) Relative expression of *PRUPE.1G169100* and *PRUPE.1G169200* obtained by comparative RNA-Seq profile during the inoculation of ‘Huyou018’.

**Supplementary Figure 10** The consistent resistant performance of ‘Sunfre’ at three-year-old and eight-year-old ‘Sunfre’.
