# Supplementary figures and images for: Single Nucleotide Polymorphism Detection for Peach Gummosis Disease Resistance by Genome-Wide Association Study
Source: Front Plant Sci. 2022 Feb 7;12:763618. doi: 10.3389/fpls.2021.763618 (PMC8858797; doi:10.3389/fpls.2021.763618)

## Slide 1
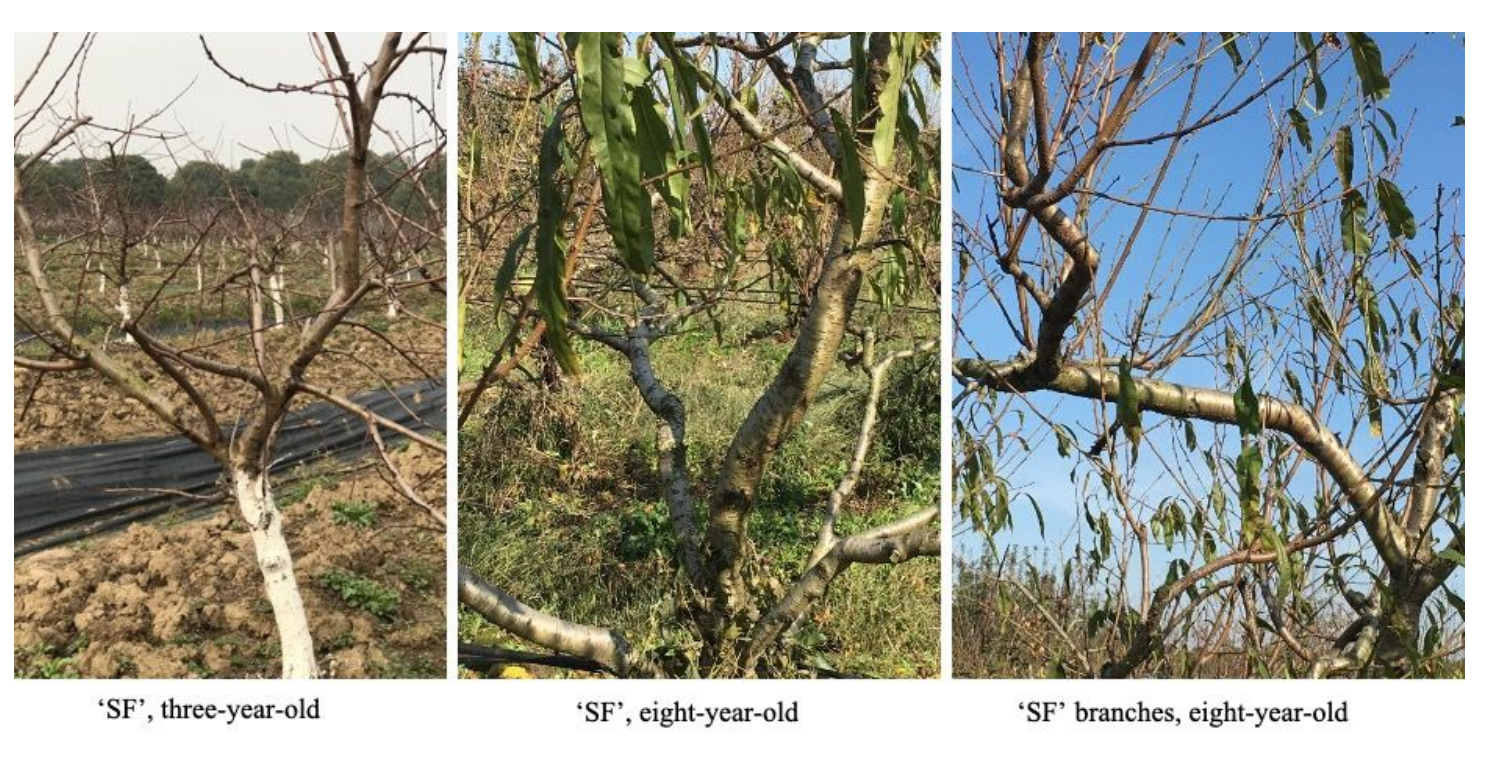

Supplement: Supplementary file 1 [file Data_Sheet_1.ZIP › Supplementary figures and tables/Supplementary Figure 10.pptx]

## Slide 1
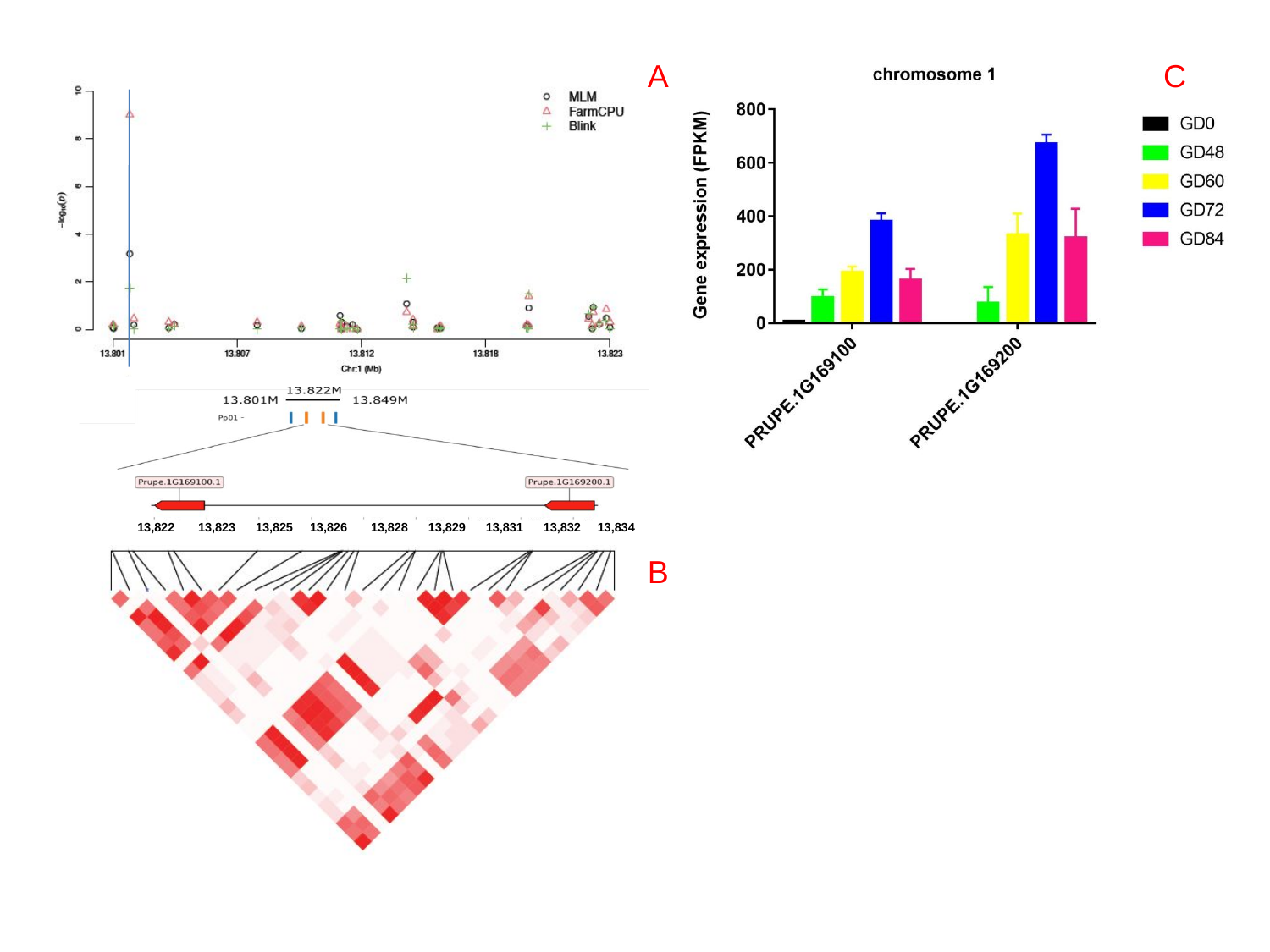

13,822 13,823 13,825 13,826 13,828 13,829 13,831 13,832 13,834
A
B
C

Supplement: Supplementary file 1 [file Data_Sheet_1.ZIP › Supplementary figures and tables/Supplementary Figure 9.pptx]

## Slide 1
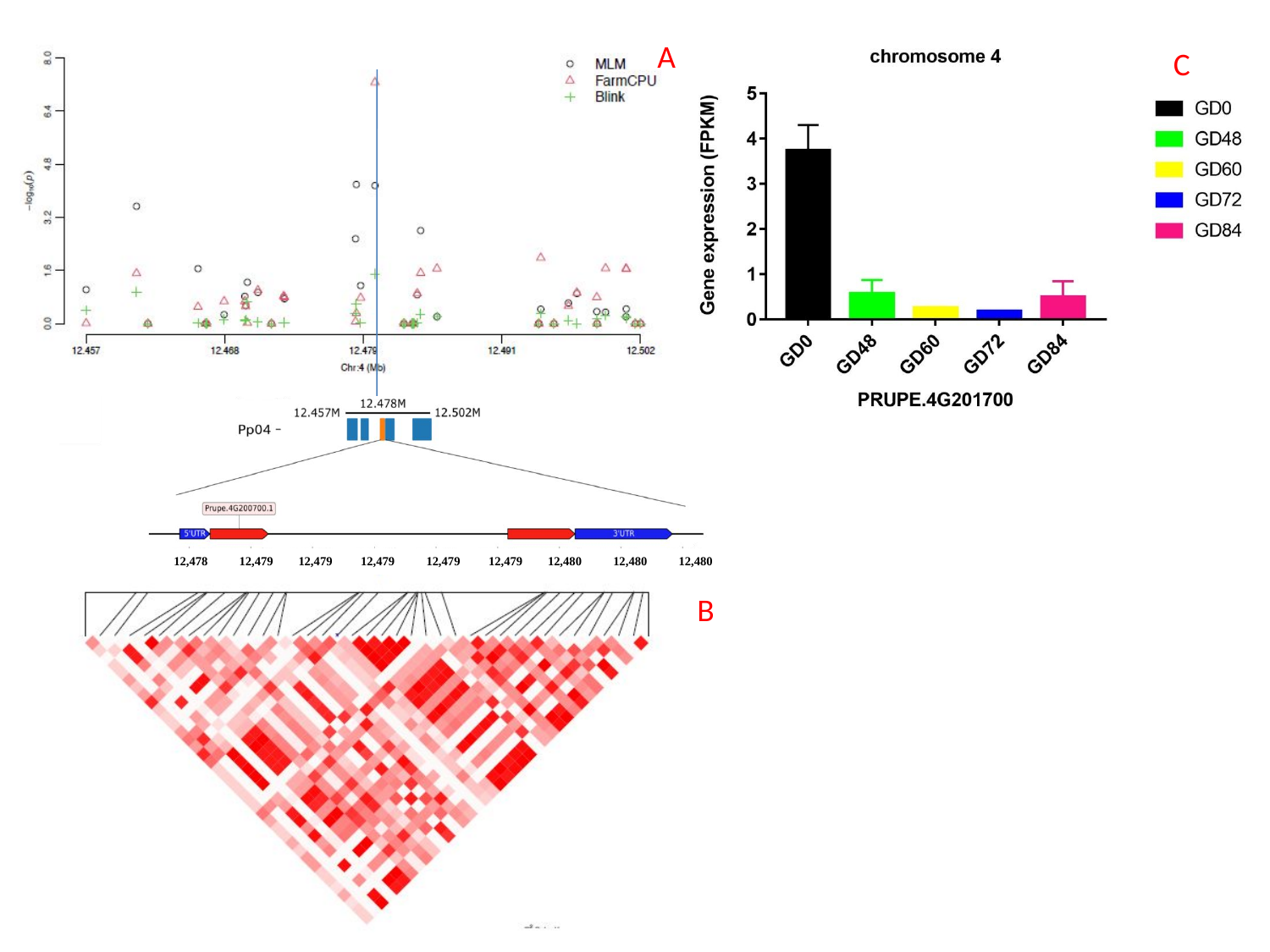

A
12,478 12,479 12,479 12,479 12,479 12,479 12,480 12,480 12,480
B
C

Supplement: Supplementary file 1 [file Data_Sheet_1.ZIP › Supplementary figures and tables/Supplementary Figure 8.pptx]

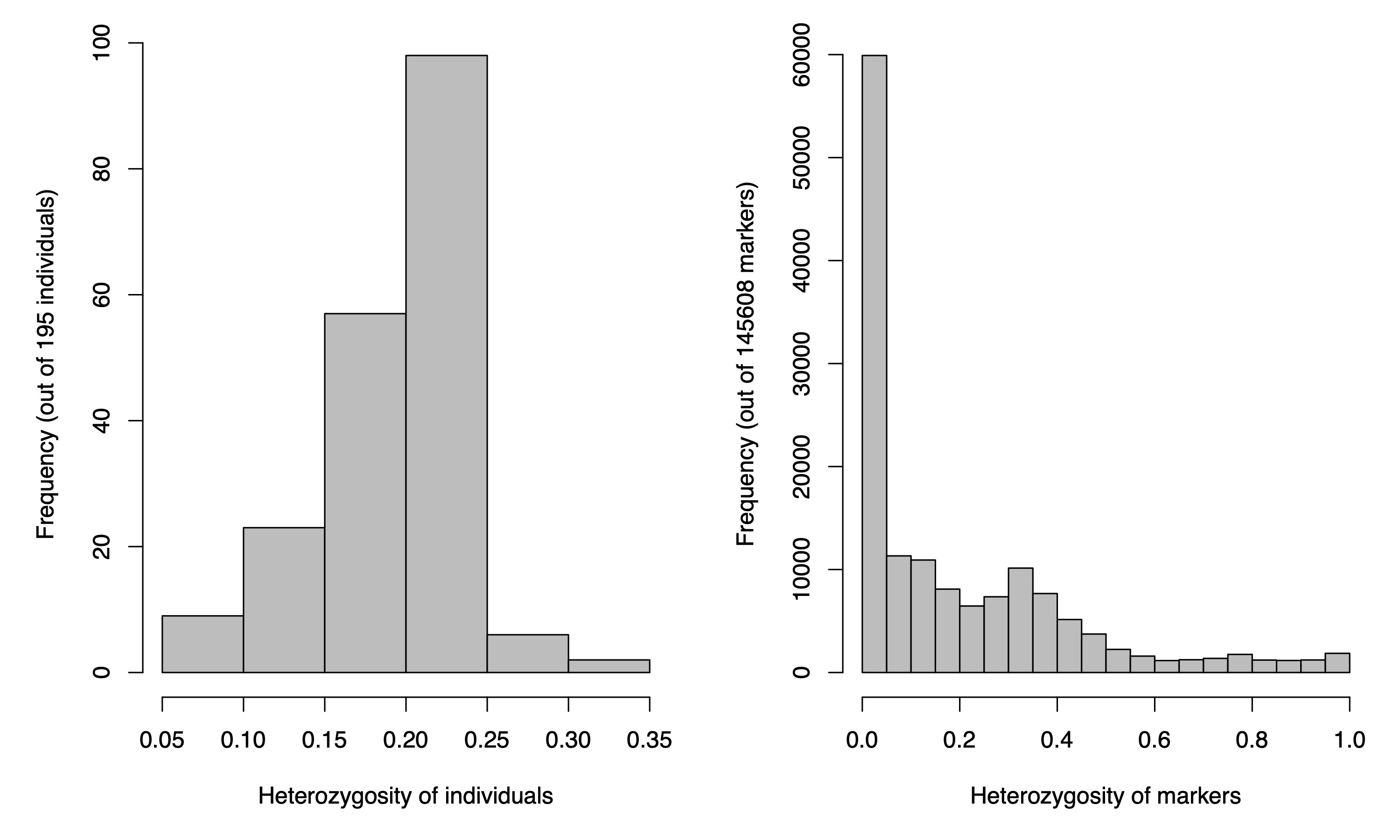

Supplement: Supplementary file 1 [file Data_Sheet_1.ZIP › Supplementary figures and tables/Supplementary Figure 3.JPEG]

## Slide 1
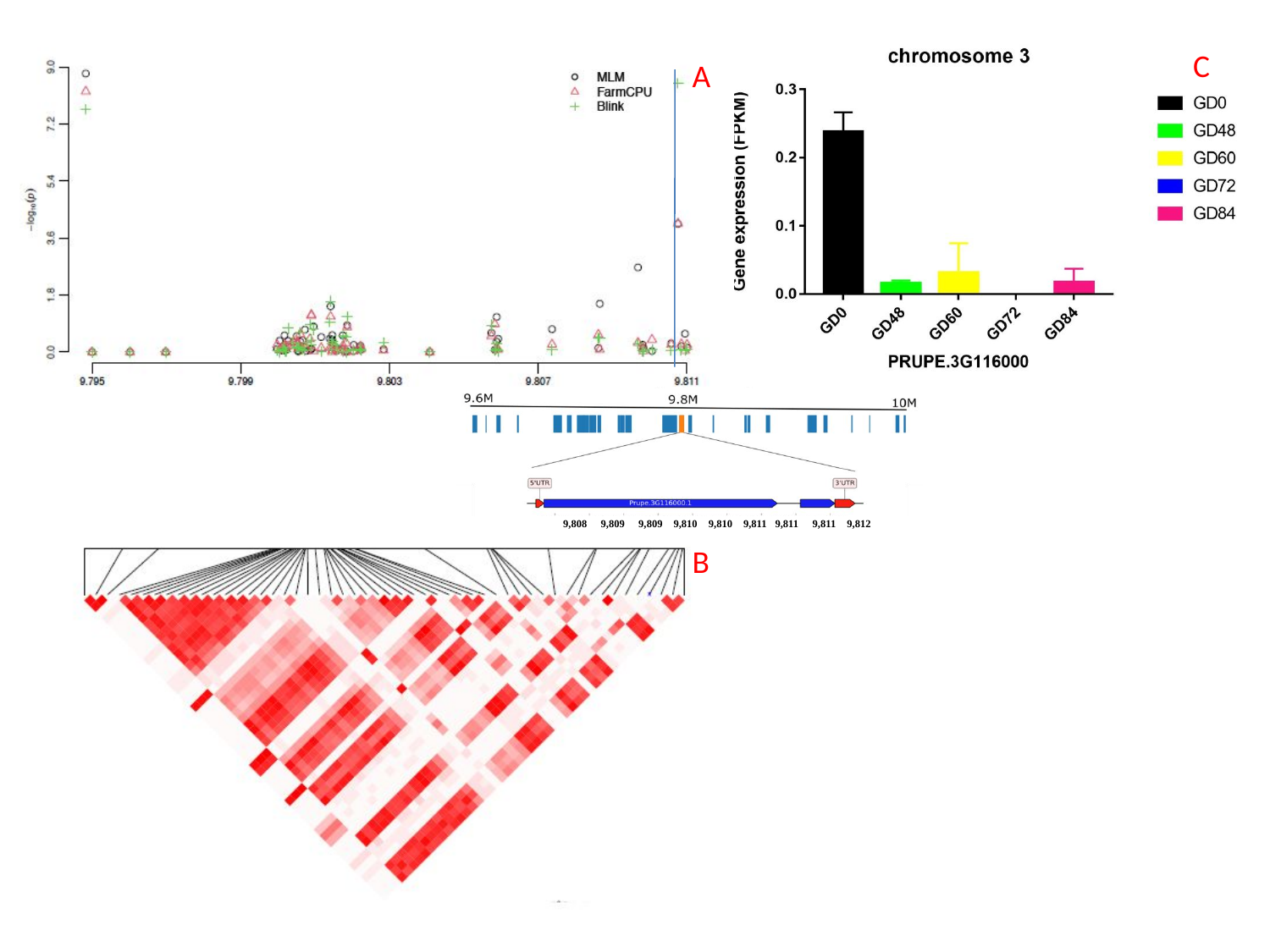

Gene expression (FPKM)
A
9,808 9,809 9,809 9,810 9,810 9,811 9,811 9,811 9,812
B
C

Supplement: Supplementary file 1 [file Data_Sheet_1.ZIP › Supplementary figures and tables/Supplementary Figure 7.pptx]

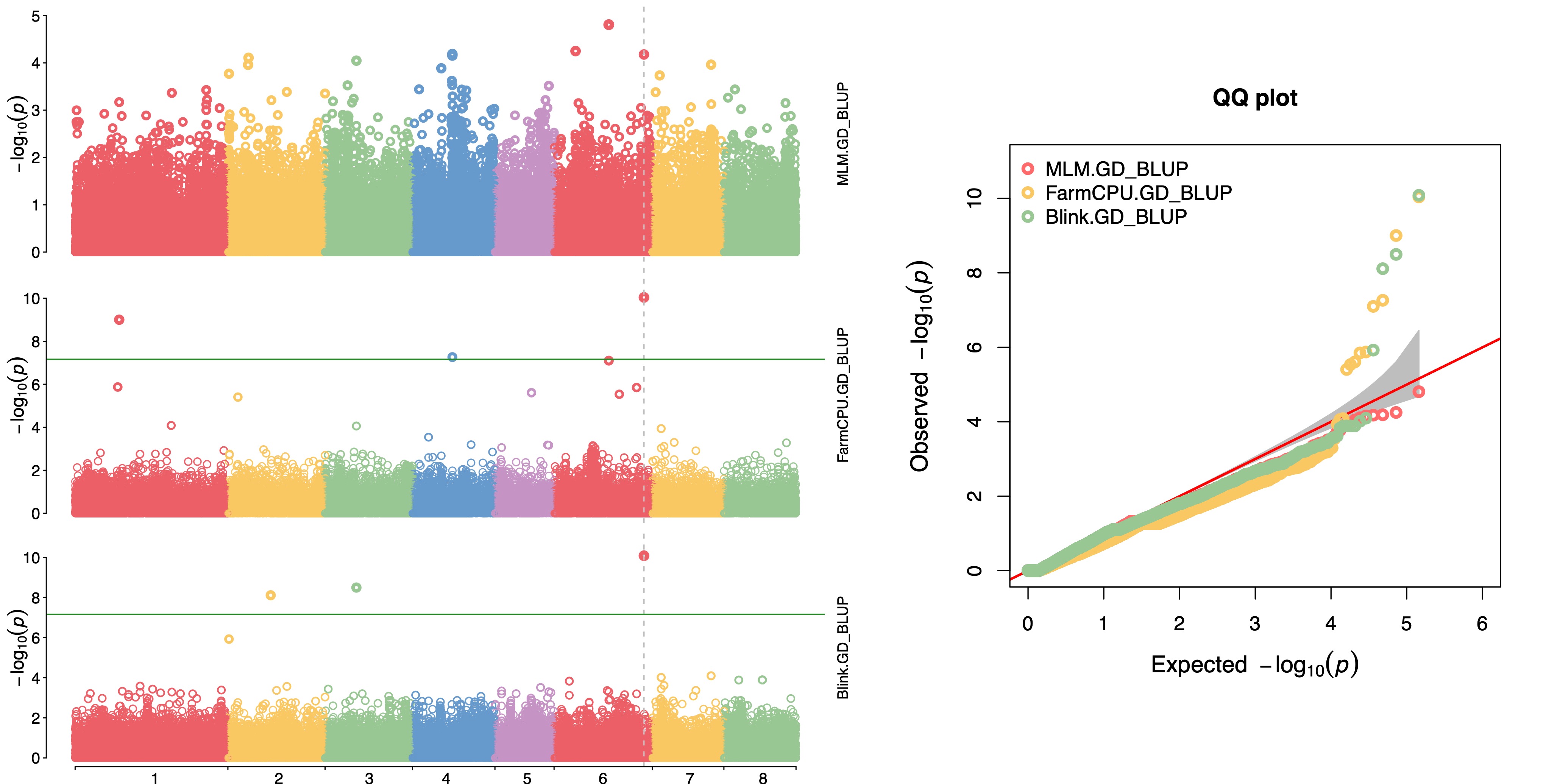

Supplement: Supplementary file 1 [file Data_Sheet_1.ZIP › Supplementary figures and tables/Supplementary Figure 5.jpg]

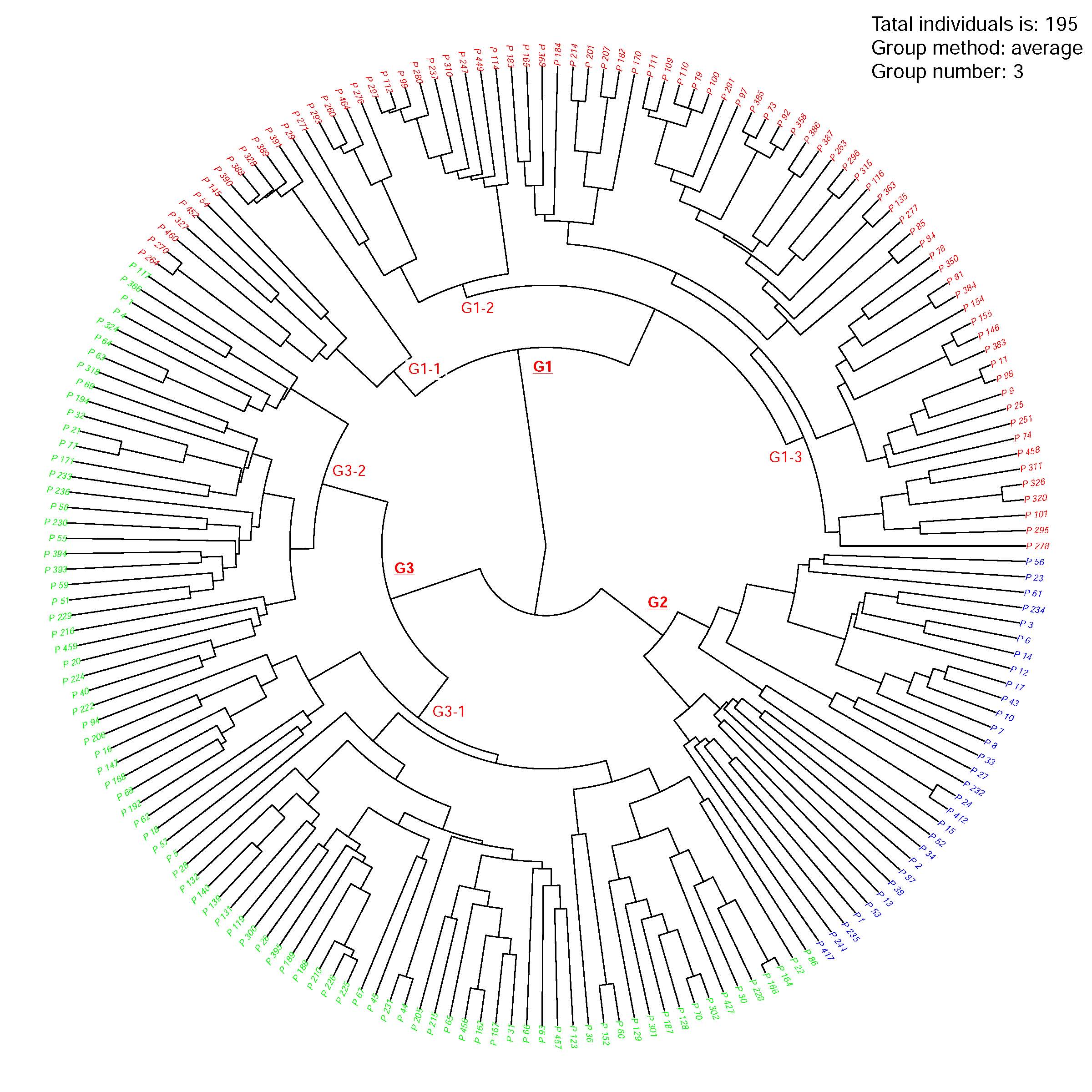

Supplement: Supplementary file 1 [file Data_Sheet_1.ZIP › Supplementary figures and tables/Supplementary Figure 4.jpg]

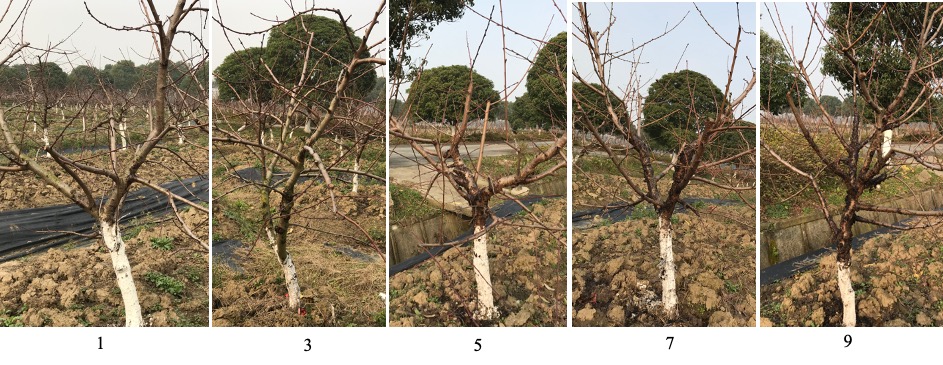

Supplement: Supplementary file 1 [file Data_Sheet_1.ZIP › Supplementary figures and tables/Supplementary Figure 1.jpg]

## Slide 1
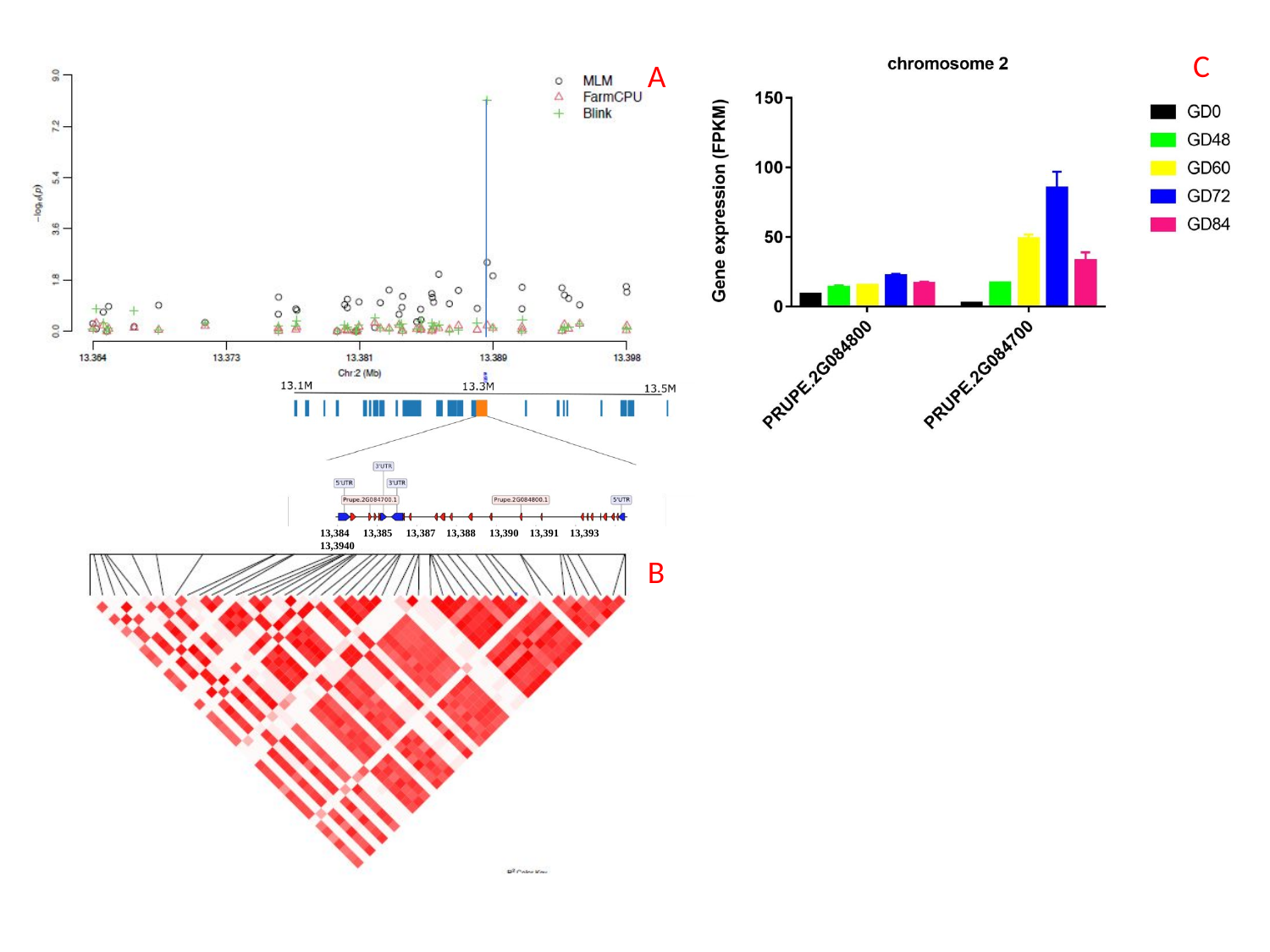

A
13,384 13,385 13,387 13,388 13,390 13,391 13,393 13,3940
B
C

Supplement: Supplementary file 1 [file Data_Sheet_1.ZIP › Supplementary figures and tables/Supplementary Figure 6.pptx]

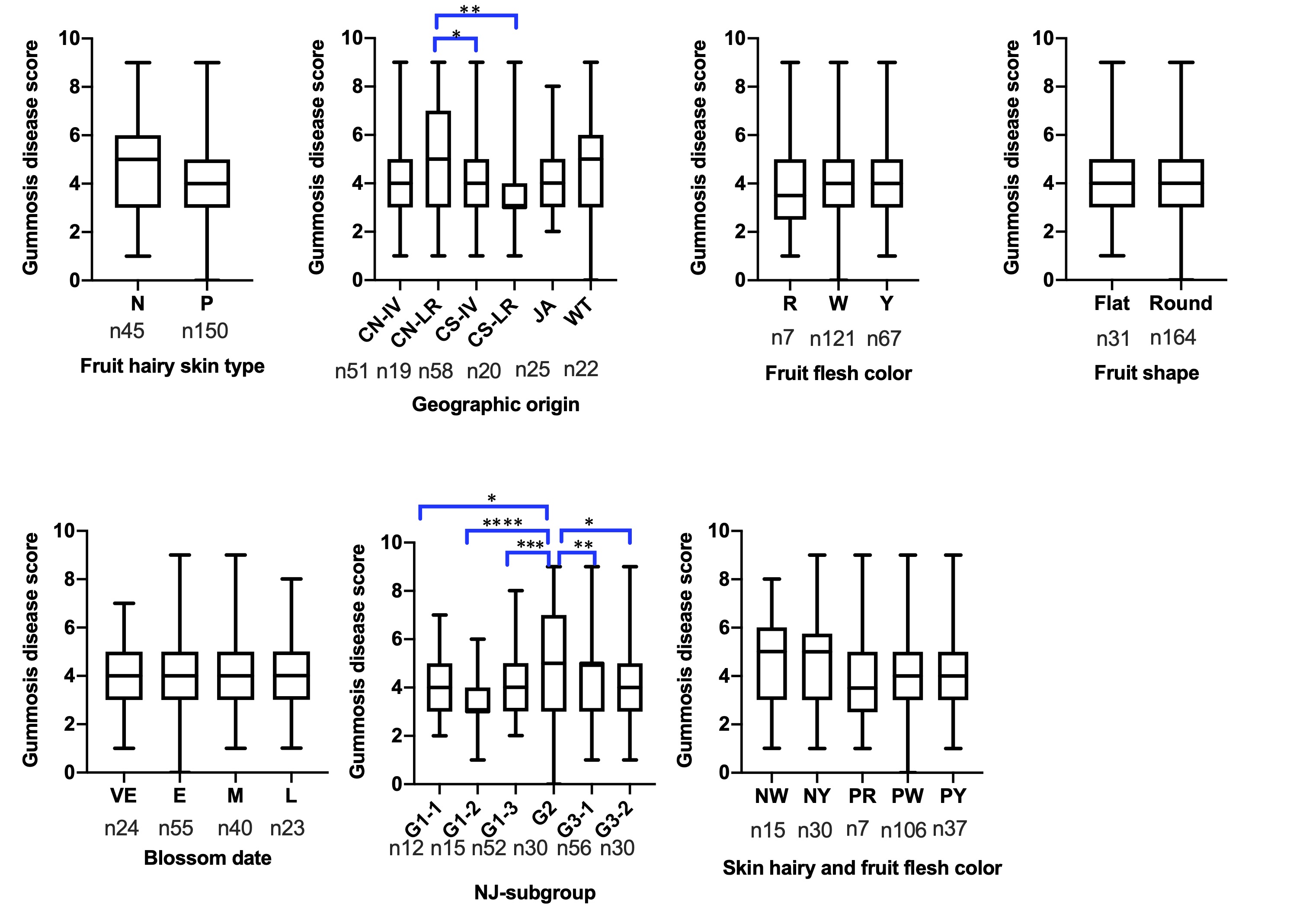

Supplement: Supplementary file 1 [file Data_Sheet_1.ZIP › Supplementary figures and tables/Supplementary Figure 2.jpg]
